# Water Activity as an Indicator for Antibody Storage Stability in Lyophilized Formulations

**DOI:** 10.1021/acs.molpharmaceut.4c01106

**Published:** 2025-01-14

**Authors:** Maximilian Zäh, Christoph Brandenbusch, Sebastian Groël, Gerhard Winter, Gabriele Sadowski

**Affiliations:** † Laboratory of Thermodynamics, Department of Biochemical and Chemical Engineering, TU Dortmund University, Emil-Figge-Street 70, Dortmund 44227, Germany; ‡ Department of Pharmacy, LMU Munich, Chair of Pharmaceutical Technology and Biopharmaceutics, Butenandtstr. 5, Munich 81377, Germany

**Keywords:** molecular, lyophilized, interactions, water, study, design, activity, glass-transition, long-term, stability, formulations., coefficient, residual, content, excipient, using, formulation, high, moisture

## Abstract

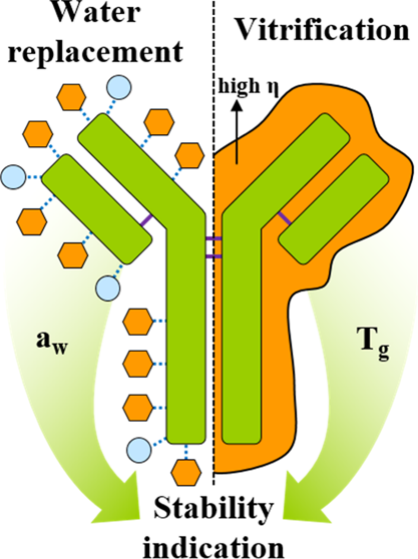

Lyophilization remains a key method for preserving sensitive biopharmaceuticals such as monoclonal antibodies. Traditionally, stabilization mechanisms have been explained by vitrification, which minimizes molecular mobility in the lyophilized cake, and water replacement, which restores molecular interactions disrupted by water removal. This study proposes a novel design strategy that combines water activity and glass-transition temperature as the main indicators to predict long-term stability in lyophilized formulations. The water activity, calculated as the product of water activity coefficient and (residual) water content, serves as a mutual indicator of molecular interactions and influence of residual water content in the lyophilizate. By predicting beneficial excipient combinations through activity coefficient calculations using the perturbed-chain statistical association fluid theory model and calculating *T*_g_ using the Gordon–Taylor equation, the study identifies favorable excipient systems, such as sucrose/ectoine mixtures, providing formulation windows that offer broad stability ranges. The approach was validated with stability studies, confirming that formulations within a water activity range of 0.025–0.25 exhibit high (long-term) stability. This work advances formulation development by integrating water-excipient interactions and residual moisture content into a predictive model, moving beyond traditional empirical methods and offering a robust pathway to the design of stable biopharmaceutical formulations. This makes it possible to achieve high/favorable water activities despite low residual moisture (thus, high glass-transition temperatures) with plausible excipient concentrations and combinations.

## 1 Introduction

Lyophilization, or freeze-drying, still is one of the gold standards in the storage and preservation of sensitive biopharmaceutics, e.g., monoclonal antibodies (mAb) or viral vectors. Stabilization within lyophilized biopharmaceutical formulations during drying and storage is achieved by the addition of excipients or excipient combinations and adjustment of the dry matrix to a defined residual moisture. The selection of excipients, and the decision on the residual moisture after drying, is usually based on empirical rules and personal expertise.^1−3^ Generally accepted, two stabilization mechanisms govern the (long-term) stability of biopharmaceuticals and biologics after lyophilization.

1 The vitrification theory attributes the stabilization of biopharmaceutics in lyophilized formulations to kinetic inhibition of molecular interactions. High viscosities in the lyophilized formulation slow down molecular interactions and thus delay degradation reactions and prolong shelf life. Specifically important in this context are the glass-transition temperature *T*_g_ and relaxation phenomena within the lyophilized formulation.^4−8^
2 The water-replacement theory attributes the stabilization of biopharmaceutics in lyophilized formulations to molecular interactions in the formulation matrix. The excipients reinstitute interactions with the biopharmaceutics formerly being governed by water molecules in the liquid (solution) formulation. Specifically, properties like the hydrogen bonding between excipients and biopharmaceutics can be connected to this stabilization principle.^9−15^

Many former investigations have shown that stabilization of biopharmaceuticals is not exclusively explained by one of the two mechanisms. For example, macroscopic (cake) stability can be compromised by an insufficiently high glass-transition temperature eventually even leading to collapse of the amorphous cake, although the excipients present are proven to be beneficial regarding their water-replacement properties.^10,16^ Vice versa, a high glass-transition temperature and thus low reaction rates and slow relaxations would not necessarily stabilize the biopharmaceutical in the lyophilized formulation when water replacement would be provided insufficiently.^2,16^ In order to design a stabilizing formulation for biopharmaceuticals, attention must, therefore, be paid to the interplay of both stabilization mechanisms.

Indicators for the stabilization of biopharmaceuticals via vitrification are high frequency β-relaxations in the lyophilizate.^13^ It was shown that the mean-squared displacement of hydrogen atoms is correlated with the chemical degradation rate.^6^ A possible indicator for water replacement is hydrogen bonds being reinstitated by excipients depending on the molar/mass ratio of excipient/biopharmaceutical. Advantageous ratios for the hydrogen bonding and the stabilization of biopharmaceuticals have been identified experimentally for various excipients. Generally, interexcipient differences cannot be accounted for, and no general statement is possible.^10,12,13^ Aside from these sole indicators, reliable methods such as the ReFOLD assay for proteins published by Svilenov and Winter that provide efficient and rapid information on stabilizing capabilities of different formulations^17^ have been developed lately. Although offering valuable information into the stabilizing capabilities of lyophilized formulations, the disadvantage of all mentioned methods and indicators is that each formulation has to be analyzed separately (high-throughput screening experiments). It is not possible to make predictive statements about potential stabilization capabilities, especially at different residual moisture values or for new combinations of excipients. Aside from the stabilization potential of certain excipients and excipient combinations, the impact of residual moisture on the (long-term) formulation stability is often considered only a side note. Heuristics often suggest that, depending on the excipients used, drying is typically carried out to below 3 w %, preferably 2% water/residual moisture.^18^ This is meant to ensure that the glass-transition temperature of the lyophilizate is sufficiently high to achieve low/zero molecular mobility in the lyophilized solution.^19^ It has been controversially discussed for many years but has now been confirmed that lyophilized formulations can be “over-dried” and, as a result, would then provide poor protein stabilization. As a rule of thumb, water concentrations of well below 0.1 wt % should therefore be avoided. However, no universal beneficial residual moisture level for formulations was identified yet.^20^

In general, predictive and reliable strategies for the design of lyophilized formulations are highly desirable, aside from classical pharma-proteins such as monoclonal antibodies, especially in the context of evolving biologics entities such as, e.g., vaccines and viral vectors.^21,22^

Within this work, we thus propose a novel thermodynamics-based design strategy, combining water activity a_w_ and *T*_g_ as determinants for a quantitative and holistic access to the (long-term) stability in lyophilized formulations. Water activity herein can be regarded as a measure/indicator for water availability both in the initial liquid formulation as well as the lyophilizate after freeze-drying. This free unbound water, which is not bound to any surfaces or occupied in molecular interaction/chemical bounds,^23,24^ can participate in the stabilization of biopharmaceutics. Water activity is known as a crucial factor influencing the degradation of antibodies and the chemical stability of the amorphous phase in which the antibody is preserved.^25^ Water activity is defined as the product of the water activity coefficient γ_w_ and the water mole fraction *x*_w_ (which is identical with residual moisture) in the liquid/amorphous/dried phase.
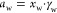
1

The water activity coefficient γ_w_ is a measure for the (molecular) interactions of water with its surrounding molecules and thus nonidealities in solution. It is highly affected by the excipients used, as well as their concentrations. Water activity therefore contains combined information on the impact of excipients and excipient combinations in the amorphous phase as well as the effect of residual moisture in the amorphous phase. If aiming for a certain value of water activity, the only levers are the water activity coefficient, which is influenced by the type and concentration of excipients, as well as the residual moisture. Calculation of the water activity coefficients can easily be achieved using the equation of state perturbed-chain statistical association fluid theory (PC-SAFT).^26,27^

The second determinant considered in our approach is *T*_g_, which is also influenced by the excipient choice and the residual moisture.^28^ Following the vitrification theory, a lyophilized formulation must have a glass-transition temperature *T*_g_ that is much higher than the storage temperature to be kinetically frozen, which dramatically slows down degradation reaction kinetics and molecular mobility. This also prevents mechanical collapse of the lyophilized formulation to take place during storage, preserving a macroscopically appealing product. Within our design strategy, we use the Gordon–Taylor approach (eq 2, written for ternary systems)^29^ to estimate/predict *T*_g_ of the respective excipients/excipient combinations as a function of the residual moisture.

Herein, the glass-transition temperature *T*_g_ is estimated using the pure components’ glass-transition temperatures *T*_g,i_ and the respective weight fractions w_i_ of the three components, that is, water and two excipients. The two Gordon–Taylor constants *k*_1_ and *k*_2_ are fitted to the glass-transition temperatures of the respective binary excipient/water system.^30^
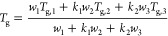
2

As both values, water activity *a*_w_ and *T*_g_, are thus coupled by residual moisture and excipient type used, finding a formulation that exhibits promising water activity and kinetic stabilization through a sufficiently high *T*_g_ requires simultaneous optimization. In the first step, the water activity coefficient of the excipient composition was optimized and maximized to allow the widest possible design range for the residual moisture. Subsequently, Gordon–Taylor was used to calculate the range of residual moisture that enabled kinetic stabilization. This approach was tested on a sucrose/ectoine model system. Compositions that were predicted by this approach to be advantageous were identified, and their selection was validated with regular stability studies.

## 2 Materials and Methods

### 2.1 Chemicals

Sucrose with a purity of ≥99.5%, dl-proline with a purity of ≥99%, and l-arginine with a purity of ≥99 were purchased from Sigma-Aldrich Co. LLC (Hamburg, Germany). l-Histidine monohydrochloride monohydrate with a purity of ≥99% was purchased from Thermo Fisher Scientific (Darmstadt, Germany). Polysorbate 20 was purchased from Croda Inc. (Snaith, UK). Ectoine with a purity of ≥98.5% was provided from bitop AG (Witten, Germany). Water from a Sartorius purification system was used to prepare the samples for analysis. The studied IgG_1_ antibody had a size of around 145 kDa.

### 2.2 Stability Testing

After the water activity of formulations from the literature was modeled using PC-SAFT and correlated with the corresponding stability data, this correlation was expanded and consolidated with further stability studies. For this purpose, advantageous excipient combinations were identified and subjected to stability studies.

#### 2.2.1 Preparation and Lyophilization

Before use, the antibody solution was purified with an KTA purification system using a Sepharose HiTrap SP column from Cytiva (Marlborough, USA). The buffer was exchanged with a 20 mM histidine buffer at pH 5.5 and 0.2% PS20 using a Minimate crossflow filtration unit with a poly(ether sulfone) (PES) membrane with a molecular cutoff of 30 kDa from Pall Corporation (New York, USA). To achieve the desired antibody concentrations of 10 mg/mL, a NanoDrop 2000 UV photometer from Thermo Fisher Scientific (Waltham, USA) was used, and the antibody solution was diluted accordingly. Excipient stock solutions were prepared using the same histidine buffer as for the antibody stock solution. Prior to mixing the various stock solutions, filtration using a 0.22 μm PES Sartolab RF vacuum filter unit from Sartorius (Göttingen, Germany) was performed for all stock solutions. To prevent moisture ingress from the used vials and stoppers during storage, the 2 R vials from MGlas (Münnerstadt, Germany) and 13 mm Lyo Nova Pure RS 1356 4023/50 G stoppers from West Pharmaceutical Services (Paxton, USA) were dried at 105 °C for 6 h and at 80 °C for 8 h, respectively. Stock solutions were mixed to achieve desired excipient concentrations; see Table 1. After that, 1 mL of formulation was filled into the vials and semistoppered. Two lines of buffer-filled vials were placed on the lyophilization rack as a radiation shield around the sample vials.

**Table 1:** Concentration in mg/mL of the Antibody and Used Excipients

antibody	sucrose	ectoine	arginine	proline
**10**	80	0	0	0
**10**	60	20	0	0
**10**	40	40	0	0
**10**	60	0	20	0
**10**	60	0	0	20

The prepared formulations were lyophilized following the program listed in Table 2. For formulations containing arginine/proline, lyophilization was performed with and without an additional annealing step in an Epsilon 2-6D LCSplus by Martin-Christ (Osterode am Harz, Germany). Remaining formulations were dried using an FTS LyoStar from SP Scientific (Stone Ridge, USA). After lyophilization, the vials were stoppered at 600 mbar and crimped with flip-off seals. Samples were stored/stressed at 25 and 40 °C, and analysis was performed after drying and after 9 months

**Table 2:** Temperature and Pressure Profile for the Lyophilization Processes^a^,^b^

step	temperature/°C	time/h	pressure/mbar
loading	25		1000
freezing	–50	1.5	1000
*annealing	–15	8	1000
primary drying	–20	48	0.06
^+^secondary drying	40	6/12	0.06

a *For the runs including the annealing step, the temperature was reduced again to −50 °C after the duration of annealing. ^+^The lower duration was used to create samples with high residual moisture.

b All heating/cooling ramps were 1 K/min.

#### 2.2.2 Karl Fischer Titration

Residual moisture after lyophilization was determined with Karl Fischer headspace titration. An AQUA 40.00 titrator from ECH Elektrochemie Halle GmbH (Halle, Germany) was used. A sample mass of around 20 mg was prepared under a dry atmosphere with <10% relative humidity. Water in the samples was then evaporated and transferred to the titration chamber for moisture content determination.

#### 2.2.3 Differential Scanning Calorimetry

Glass-transition temperatures were determined using difference scanning calorimetry (DSC) using a Q2000 DSC with an attached RCS90 temperature control unit from TA Instruments (Eschborn, Germany). Between 5 and 10 mg of lyophilized sample was hermetically sealed in aluminum pans and heated from 0 to 200 °C with a heating ramp of 5 K/min. *T*_g_ was interpreted as the infliction point of the heat flow using the software TA universal analysis.

#### 2.2.4 Powder X-ray Diffraction

To ensure the maintained amorphicity of the lyophilized samples, powder X-ray diffraction (PXRD) using a Miniflex 600 from Rigaku (Tokyo, Japan) with a Cu Kα anode in reflection mode with a tube voltage of 40 kV and a current of 15 mA was used. The scanning rate was 5° 2θ/min from 5° to 35° 2θ.

#### 2.2.5 Size-Exclusion Chromatography

The monomer content of the antibody formulations was determined by using a Q1260 Infinity II Quaternary size-exclusion chromatography system (SEC) from Agilent Technologies (Santa Clara, USA). It includes pumping, degassing (G7111B), autosampling (G7129), UV–vis absorption (G7115A), RI-refraction (G7162A), and light scattering using the miniDAWN from Wyatt Technology Corporation (Santa Barbara, USA). For separation, a SEC Superdex 200 Increase 10/300 GL column from Cytiva (Marlborough, USA) was used. UV analysis was performed at a 280 nm wavelength. Prior to analysis, lyophilized samples were reconstituted with 910 μL of purified water. Samples were then centrifuged at 10,000 rpm for 10 min using a 5425 centrifuge from Eppendorf (Hamburg, Germany). For SEC analysis, 10 μL was injected. Mobile phase was a 50 mM phosphate buffer at pH 7 with a flow rate of 1 mL/min. Astra V.7.3.2 from Wyatt Technology Corporation (Santa Barbara, USA) was used to determine the mass fractions of antibody monomer. From the antibody monomer content, antibody (mAb) monomer retention was calculated.
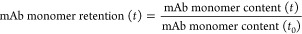
3

#### 2.2.6 Nano Differential Scanning Fluorimetry

Thermal stability was analyzed using nano differential scanning fluorimetry (nanoDSF) using the Prometheus NT.48 from NanoTemper (Munich, Germany). Reconstituted samples (see Section 2.2.5) were put into capillaries and heated from 20 to 90 °C with a heating ramp of 2 K/min. The fluorescence ratio F350/F330 was used to determine the unfolding temperature T_unfold_ and the aggregation temperature *T*_agg_.

## 3 Modeling Using PC-SAFT

The activity coefficients necessary for the calculation of the water activity are derived from the residual Helmholtz energy *A*^res^. *A*^res^ is calculated with the PC-SAFT.^26,27^ In this process, *A*^res^ is composed of different fractions. *A*^hard-chain^ accounts for hard-chain repulsions, *A*^dispersion^ accounts for associative interactions, and *A*^association^ accounts for hydrogen bonding.
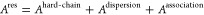
4For the calculation of *A*^res^, each molecule is described as a chain of *m*_*i*_^seg^ segments, with each segment having a diameter σ_*i*_. In addition, the dispersion–energy parameter *u*_*i*_/*k*_B_, the association–energy parameter *e*^*AiBi*^/*k*_B_, and the association volume *k*^*AiBi*^ are considered. These quantities are calculated using Berthelot–Lorentz^31^ mixing rules, introducing an adjustable binary interaction parameter *k*_*ij*_.
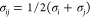
5
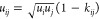
6

The binary interaction parameter *k*_*ij*_ can be temperature-dependent with a constant value *k*_*ij*,0K_ at 0 K and a temperature slope *k*_*ij*,*T*_.
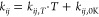
7

For the calculation of the association energy and volume, mixing rules of Wolbach and Sandler^32^ were used.
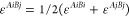
8
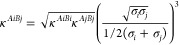
9

The PC-SAFT pure component parameters for the calculation of *a*_w_ for the data from Haeuser et al.^16^ containing cyclodextrins, recombinant human albumin, and polyvinylpyrrolidone in addition to sucrose, ectoine, arginine, and proline are listed in Table 3. To model the influence of recombinant human albumin, the pure component parameters and interaction parameters of bovine serum albumin were used due to its comparable structure.

**Table 3:** PC-SAFT Pure Component Parameters of the Substances Used in This Work

substance	*M*_i_/g mol^–1^	*m*_i_^seg^/mol g^–1^	σ_i_/Å	*u*_i_ *k*_b_^–1^/K	ε^*AiBi*^ *k*_b_^–1^/K	*k*^*AiBi*^	*N*_i_^assoc^	refs.
sucrose	342.30	0.04349	2.827	297.39	5000.0	0.100	8/8	(33)
ectoine	142.16	0.00879	5.050	530.00	3500.0	0.090	2/2	(33)
arginine	174.20	0.05688	2.657	349.71	2555.5	0.039	3/1	(34)
proline	115.13	0.06064	2.548	289.72	5527.8	0.036	1/1	(34)
cyclodextrin	1493	0.02878	2.933	244.53	5000.0	0.100	21/21	(35)
recombinant human albumin/bovine serum albumin	66,000	0.06235	2.600	344.58	1892.6	1.000	176/153	(36)
polyvinylpyrrolidone	1,220,000	0.04070	2.710	205.60	0.0	0.020	10,977/10,977	(37)
water	18.02	1.20147	a	353.94	2425.7	0.045^b^	1/1	(34)

a σ = 2.7927 + 10.11·exp(−0.01775·T) – 1.417·exp(−0.01146·T).

b Typo in ref (34).

For the calculation of the water activity, binary interaction parameters were used and are listed in Table 4.

**Table 4:** PC-SAFT Binary Interaction Parameters Used in This Work

substances	*k*_ij,T_/K^–1^	*k*_ij,0K_	refs.
sucrose/water	0.000256	–0.1134	(38)
ectoine/water	0.000578	–0.1696	(39)
arginine/water		–0.0145	(33)
proline/water		–0.0699	(33)
cyclodextrin/water		–0.0758	(35)
recombinant human albumin/bovine serum albumin/water		–0.0716	(36)
polyvinylpyrrolidon/water		–0.1483	(40)

## 4 Results and Discussion

### 4.1 Applicability of Water Activity as a Stability Criterion in Lyophilized Formulations

In order to evaluate the applicability of water activity as stability criterion in lyophilized formulations, we first investigated a possible correlation using available literature data.^16^ Stability data was selected from mAb formulations that had different excipient compositions with largely uniform residual moisture. Water activity of the respective formulations was calculated using PC-SAFT, as described in Section 3. The results are listed in Table 5. Detailed compositions of respective formulations are given in the appendix.

**Table 5:** Calculated Water Activity and mAb Monomer Retention after 9 Months of Storage at 40 °C for Literature Data^a^

#	*a*_w_	mAb monomer retention	#	*a*_w_	mAb monomer retention
1	4.81 × 10^–5^	0.86	**13**	9.03 × 10^–3^	0.97
2	4.81 × 10^–5^	0.84	**14**	9.03 × 10^–3^	0.96
3	7.41 × 10^–5^	0.91	**15**	9.56 × 10^–3^	0.97
4	7.41 × 10^–5^	0.89	**16**	9.56 × 10^–3^	0.96
5	1.18 × 10^–4^	0.98	**17**	9.84 × 10^–3^	0.99
6	1.77 × 10^–4^	0.97	**18**	9.84 × 10^–3^	0.99
7	2.75 × 10^–4^	0.95	**19**	1.13 × 10^–2^	0.97
8	2.75 × 10^–4^	0.93	**20**	1.13 × 10^–2^	0.96
9	6.33 × 10^–3^	0.99	**21**	1.27 × 10^–2^	0.97
10	6.33 × 10^–3^	0.99	**22**	1.27 × 10^–2^	0.97
11	6.34 × 10^–3^	0.99	**23**	2.32 × 10^–1^	1.00
12	6.34 × 10^–3^	0.99	**24**	2.42 × 10^–1^	0.99

a Detailed compositions are given in the appendix.

Considering monomer retention as a function of water activity delivers a clear correlation (see Figure 5) with respect to (long-term) formulation stability. Formulations with very low water activity values show a significant loss in monomer content (if stored at 40 °C for 90 days) of up to 16% (e.g., no. 2 in Table 5, *a*_w_ = 0.000048). In a water activity range between *a*_w_ = 0.000633 and *a*_w_ = 0.0127, monomer retention of over 98% could be achieved after samples were stored for 90 days at 40 °C. The highest and almost complete monomer retention (>99.9%) was observed at water activities of 0.23 (formulation #23 in Table 5) and 0.24 (formulation #24 in Table 5).

The results clearly suggest that based on the data analyzed, the water activity of a formulation should be higher than 0.025 if a monomer retention of >97% is desired and higher than 0.23 in order to allow for the best (long-term) stabilization of the antibodies (>99.9% monomer retention). It is crucial to avoid water activities below 0.000275, as (based on the formulations investigated) this will lead to significant monomer loss during long-term storage. It has to be mentioned that the values of 0.025 and 0.23 were defined based on the available data set. With other, more comprehensive stability studies, even slightly lower water activities might be tolerable.

#### 4.1.1 Identifying the Optimal Water Activity Range

With the lower boundary (minimal value) for water activity already available through previous investigations, further investigations were performed to identify a useful upper water activity boundary. The water activity in a lyophilizate after drying can typically reach values of up to 0.4. This is because constraining meaningful dry products to an accepted residual moisture in the formulation of maximally ca. 3 wt % excludes higher water activities.

**Figure 1:**
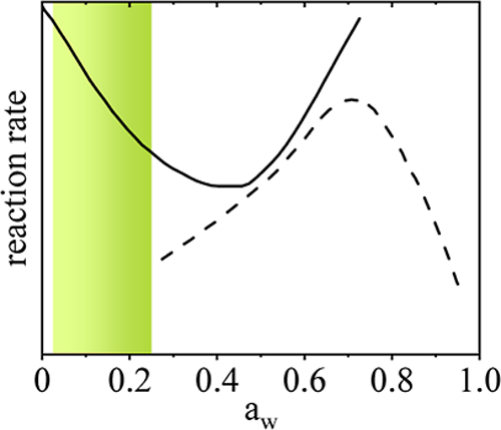
Degradation reaction rate in dependence of water activity. Solid line shows oxidation reaction rate. Dashed line shows the browning reaction rate. Data from ref (25). No absolute reaction rate was given in the reference, but relative reaction rates are shown here. The green region shows the water activity with the best anticipated stabilization.

As illustrated in Figure 1, from a general point of view, two main chemical degradation routes are typically taken into account for lyophilized formulations: oxidation kinetics can be reduced by increasing the water activity, with a minimum oxidation expected at a water activity of 0.4. However, above a critical water activity of 0.28, the Browning reaction rate constantly increases.^25^

As an optimal compromise between the two degradation reactions, the water activity should be close to the critical water activity of 0.28 in order to minimize the oxidation rate, prevent the browning reaction from taking place, and at the same time be as high as possible. This theoretical consideration overlaps well with the results from the experiments reported above in Table 5, defining the water activity window in lyophilized formulations to be in the region of 0.025 to 0.25.

### 4.2 Initial Excipient Choice Based on *a*_w_ and *T*_g_

#### 4.2.1 Identification of Promising Excipient Combinations Based on γ_w_

**Figure 2:**
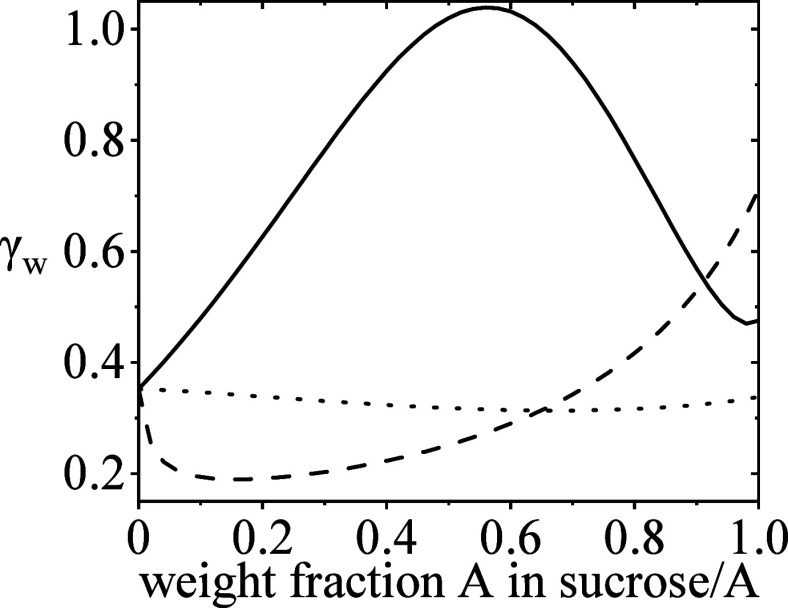
Water activity coefficient of water for excipient mixtures at infinite dilution of water. The solid line shows the combination of sucrose/ectoine. The dashed line shows the combination of sucrose/arginine. The dotted line shows the combination of sucrose/proline.

As one of the two determinants/levers for tuning a_w_ to a specific value within the desired water activity range, investigations were performed to identify excipient combinations that offer a broad tunable range in γ_w_ values depending on their composition. PC-SAFT calculations were performed, as described in Section 3. The results illustrated in Figure 2 show a promising, useful system (sucrose/ectoine, high range in γ_w_ values, mostly above the value of the single component), a non-optimal system (sucrose/arginine, low range in γ_w_ values, mostly below the value of the single components), and a “no effect” system with practically no differences in γ_w_ values over the entire ratio range of mixtures (sucrose/proline).

Systems such as sucrose/ectoine shown in Figure 2 are preferable, as the high range in γ_w_ values simultaneously allows for a high range of residual moistures to be considered while still meeting the water activity range criterion.

#### 4.2.2 Tuning *T*_g_ through the Residual Mositure

**Figure 3:**
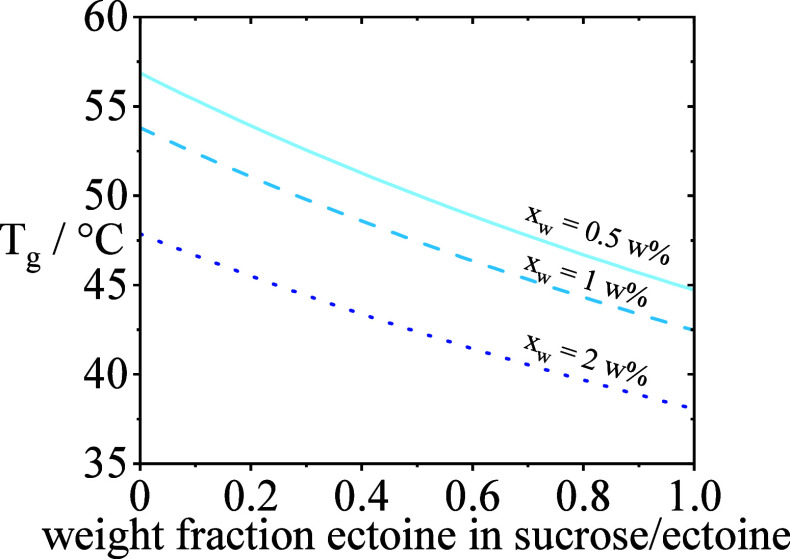
Glass-transition temperatures for mixtures of sucrose and ectoine for varying residual moistures. Light blue solid line shows *T*_g_ for 0.5 w % residual moisture. Blue dashed line shows *T*_g_ for 1 w % residual moisture. Dark blue dotted line shows *T*_g_ for 2 w % residual moisture.

The second determinant/lever investigated for tuning *a*_w_ to a specific value is the residual moisture *x*_w_, which is directly connected to *T*_g_ of the formulation. Care has to be taken to ensure that *T*_g_ of the formulation is far higher than the storage temperature (in order to ensure a glassy state/adherence to vitrification theory and avoid cake collapse). It is common knowledge that the glass-transition temperature of the lyophilizate decreases with an increase in residual moisture. That is because water has a glass-transition temperature of −137 °C. *T*_g_ of the formulations was calculated using the Gordon–Taylor equation, as described in the Introduction. Gordon–Taylor constants were fitted to the glass-transition temperatures *T*_g_’ of sucrose/water and ectoine/water, as stated in the literature.^30^ Fitting resulted in Gordon–Taylor constants of 0.311 for sucrose/water and 0.4 for ectoine/water. The effect of residual moisture on *T*_g_ is depicted in Figure 3, where an increase of 1.5 w % residual moisture lowers *T*_g_ for about 9 K for all sucrose/ectoine excipient compositions.

The Gorden–Teller equation thus allows us to calculate the (critical) residual moisture for all binary compositions of sucrose/ectoine at which the formulations/lyophilizates *T*_g_ is still above the critical value of 40 °C. This temperature was selected in order to avoid collapse of the lyophilizate during storage at *T*_storage_ = 25 °C and to ensure “zero mobility” at 2–8 °C as the regular storage temperature. For pure sucrose, the threshold *T*_g_ of 40 °C is reached at a residual moisture content of 3.4 w %. For pure ectoine, the threshold is already reached at 1.6 wt % residual moisture.

#### 4.2.3 Combining a_w_ and *T*_g_

**Figure 4:**
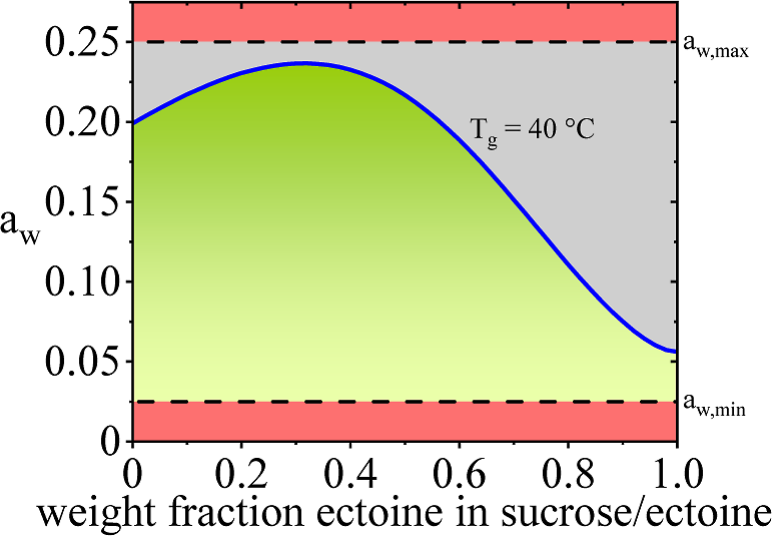
Water activity calculation sucrose/ectoine excipient mixture. The blue line shows the water activity with a constant glass-transition temperature of 40 °C. Below the blue line, glassy, and above the blue line, rubbery lyophilizates are expected. *a*_w,min_ and *a*_w,max_ depict the proposed design range of *a*_w_. The green region shows a kinetically stabilized formulation within the proposed *a*_w_ range. The gray region shows formulations where the kinetic stabilization is compromised. The red region shows where *a*_w_ is out of the proposed range.

Finally, water activity and *T*_g_ were combined/simultaneously optimized. The value for the (critical) residual moisture, as calculated in Section 4.2.2 was used and combined with PC-SAFT calculations for the activity coefficient for all (binary) formulation compositions. The resulting water activity marks the highest tolerable water activity for the respective excipient compositions, which still fulfills the *T*_g_ = 40 °C requirement (blue curve in Figure 4). For the given case, all values on this curve lie below the upper water activity boundary of 0.25, meaning that for this particular system/formulation, water activity values above the curve but below 0.25 (gray area in Figure 4) would meet the water activity criterion but fail the *T*_g_ criterion. The lower boundary for water activity remains at 0.025 (as taken from Section 4.2.1). The green area in Figure 4 thus marks the applicable formulation window that fulfills both requirements. From an application perspective, it is therefore recommended to use the 0.33 wt % ectoine in the sucrose/ectoine mixture, giving the highest flexibility in the residual water content with tolerable values between 0.24 and 2.8 w % water.

As the influence of temperature on the water activity in a formulation is small, it can be expected that predictions made at 40 °C in this regard are valid also at other storage temperatures (e.g., 25 °C and 5 °C) or vice versa. The calculation of the water activity for the temperatures 5, 25, and 40 °C is shown in the Supporting Information, and all show corresponding values.

### 4.3 Validation of Water Activity Correlation and Design Approach

In order to validate the water activity correlation and design approach described in Sections 4.1 through 4.2.3, we defined several formulations for stability testing in the water activity range between 0.01 and 0.1. In addition, a formulation with high water activity outside the proposed range was prepared to validate the upper limit for water activity. Excipient combinations containing sucrose, arginine, ectoine, and proline were selected because these excipients are widely used in the pharmaceutical industry. The excipient compositions are listed in Table 1. All formulations were dried and lyophilized, as described in Section 2.2.1. Results on residual moisture, water activity, glass-transition temperature after drying, and the monomer retention after 9 months of storage at 40 °C are given in Table 6. The residual moisture ranged between 0.31 and 0.91 w % and the water activities between 0.01 and 0.09. The glass-transition temperatures of the formulations were all above 40 °C. Formulation 8 was prepared as a negative example with a high residual moisture of 4.07 w % and a resulting water activity of 0.309, a value beyond the proposed range of a stable formulation after lyophilization. The glass-transition temperature was 26.5 °C, approximately 13.5 K below the maximum storage temperature of 40 °C.

**Table 6:** Excipient Composition, Residual Moisture, Water Activity, Glass-Transition Temperature, and Monomer Retention of Prepared Formulations after 9 Months of Storage at 40 °C

#	excipient composition	residual moisture/w %	*a*_w_	*T*_g_/°C	monomer retention
**1**	75/25 sucrose/arginine no annealing	0.4	0.012	68.9	0.99
**2**	75/25 sucrose/arginine annealing	0.3	0.010	73.3	0.97
**3**	75/25 sucrose/proline no annealing	0.3	0.014	56.7	0.98
**4**	75/25 sucrose/proline annealing	0.4	0.020	40.7	>0.99
**5**	sucrose	0.6	0.043	65.5	>0.99
**6**	75/25 sucrose/ectoine	0.7	0.071	62.9	0.99
**7**	50/50 sucrose/ectoine	0.9	0.093	66.3	>0.99
**8**	50/50 sucrose/ectoine high moisture	4.1	0.309	26.5	>0.99

Confirming the water activity calculations based on literature data performed in Section 4.1, a water activity of 0.01 leads to formulations with a monomer retention >97% after 90 days of storage at 40 °C. For water activities above 0.01, the monomer retention increased, and full retention (>99%) for formulations #1, #4, #5, #7, and #8 was found.

**Figure 5:**
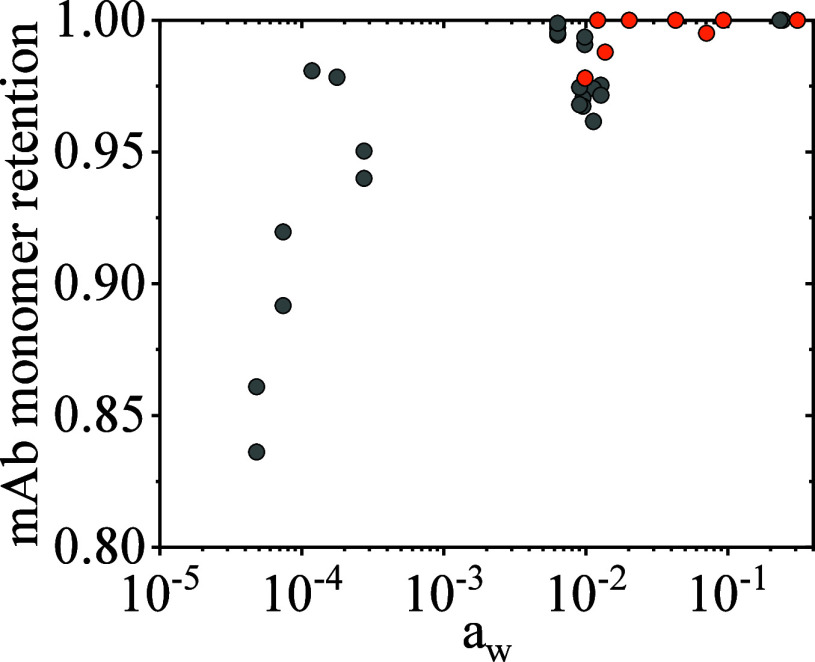
mAb monomer retention plotted over the water activity in lyophilized formulations after storage for 9 months at 40 °C. Gray points are data from ref (16), and the orange points are data from this work.

Considering all data points shown in Figure 5, the trend of high water activity leading to a better stabilization than low water activity is confirmed.

**Figure 6:**
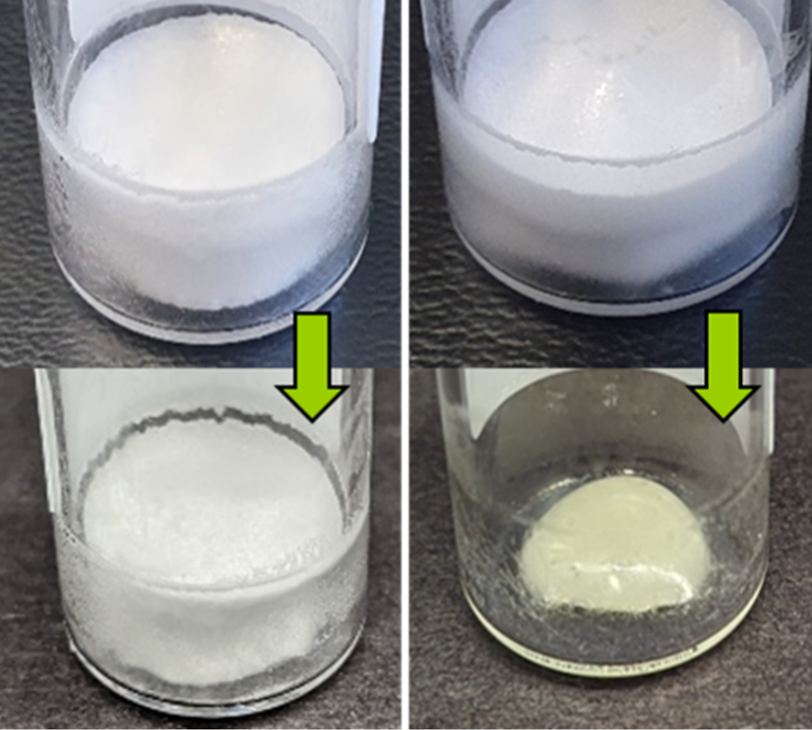
Pictures of the lyophilizates after drying (top) and after 9 months of storage at 40 °C (bottom). (left) 75/25 sucrose/ectoine mixture with a water activity of 0.09; (right) 50/50 sucrose/ectoine mixture with a water activity of 0.31.

All formulations showed little cake shrinkage after drying and cake detachment from the wall. Additionally, vial-neighboring effect was observed, resulting from intervial cooling during lyophilization, see upper pictures in Figure 6. Samples from formulation #4 showed macroscopic collapse after annealing and subsequent drying, resulting from viscous flow during annealing, as this macroscopic collapse was not observed for formulation #3 not including the annealing step. The observed collapse in formulation #4 may have led to the glass-transition temperature being much lower than the glass-transition temperature of formulation #3, although having the same excipient composition. Collapse may have led to an irregular moisture distribution, when a high residual moisture spot was analyzed using DSC as the glass-transition temperature should probably be higher and closer to the *T*_g_ of formulation #3.

No collapse or browning was observed in formulations #1–7 over the storage period, neither at 25 °C nor at 40 °C. Formulation #8 (“negative example”) collapsed after one month of storage at 40 °C. After 9 months of storage, only a collapsed, highly viscous drop remained at the bottom of the vial; see the right pictures in Figure 6. Simultaneously, the lyophilizate turned yellow, which can be attributed to browning. These two phenomena are due to the low glass-transition temperature of 23.5 °C and the water activity of over 0.28, which is the limiting water activity for a browning reaction to take place, respectively. Although complete retention of the antibody was possible at a water activity above 0.25, the recommended threshold should not be exceeded to ensure overall mechanically and chemically stable lyophilizates. Thus, high molecular mobility is not necessarily a compromising antibody stability as formulation #8 showed complete monomer retention, suggesting that stabilization by beneficial molecular interactions is the decisive factor in this case.

All formulations remained amorphous over the storage time. In addition, no change in the unfolding temperature of the antibody was observed during storage. The detailed results can be found in the appendix.

## 5 Conclusions

Within this work, we have demonstrated that the water activity can serve as a reliable indicator of antibody stability. Experimental data showed a clear correlation with monomer retention after accelerated stability testing (9 months, 40 °C). Traditional approaches typically consider only the excipient composition or residual moisture for formulation development. This work highlights that the impacts/effects of excipient choice and residual moisture can be, and should be, assessed, as it is the case using water activity. This work further indicates that no universally optimal level of residual moisture exists. The optimal level is dependent on the specific excipients used and their interactions with water. We developed an innovative design approach that facilitates the identification of promising excipients and excipient mixtures to be used for (lyophilized) formulations based on water activity calculations. By applying this approach, we were able to predict formulation compositions and formulation conditions that exhibit enhanced (long-term) stability with minimal to no experiments. Predicted formulations were validated using accelerated stability studies (9 months, 40 °C), which confirmed the validity and reliability of our design approach and, thus, the applicability of water activity as a design parameter. This work signifies an advancement in formulation development, providing a pathway to move from traditional trial-and-error methods to a more strategic and modeling-based development process. This approach also improves the likelihood of identifying stable and effective antibody formulations in the early development stages and increases the speed to find them.

## Supporting Information Available

Overview on the formulation compositions from the literature, nanoDSF data on the unfolding temperature of investigated formulations, and water activity calculations for various temperatures (PDF)

## Nomenclature

*A*_assoc_: fraction association J/mol
*A*_disp_: fraction dispersion J/mol
*A*_hc_: fraction hard chain J/mol
*A*_res_: residual Helmholtz energy J/mol
*k*_*ij*_: interaction parameters
*k*_*i*_: Gordon–Taylor constant
*m*_*i*_^seg^: number of segments mol/g
*T*: temperature K
*T*_g_: glass-transition temperature K
*T*_g_′: maximum glass-transition temperature of amorphous phase achievable through freezing K
*u*_*i*_/*k*_B_: dispersion energy K
*x*: mole fraction liquid phase mol/mol
*w*: weight fraction
*w*_g_′: weight fraction of amorphous phase at and below *T*_g_′

## Greek Symbol Description Unit

γ_*i*_: activity coefficient of component *i*
ε^*AiBj*^/*k*_B_: energy of association K
κ^*AiBj*^: association volume
ρ: density kg/m^3^
σ: segment diameter Å

## Abbreviations

(m)DSC: (modulated) differential calorimetry
PC-SAFT: perturbed-chain statistical association fluid theory

## References

[ref1] Duralliu A.; Matejtschuk P.; Stickings P.; Hassall L.; Tierney R.; Williams D. R. The Influence of Moisture Content and Temperature on the Long-Term Storage Stability of Freeze-Dried High Concentration Immunoglobulin G (IgG). Pharmaceutics 2020, 12, 303 10.3390/pharmaceutics12040303. 32230795 PMC7238084

[ref2] Breen E. D.; Curley J. G.; Overcashier D. E.; Hsu C. C.; Shire S. J. Effect of moisture on the stability of a lyophilized humanized monoclonal antibody formulation. Pharm. Res. 2001, 18, 1345–1353. 10.1023/a:1013054431517. 11683251

[ref3] Hsu C. C.; Ward C. A.; Pearlman R.; Nguyen H. M.; Yeung D. A.; Curley J. G. Determining the optimum residual moisture in lyophilized protein pharmaceuticals. Dev. Biol. Stand. 1992, 74, 255–270. 1592175

[ref4] Groel S.; Menzen T.; Winter G. Calorimetric Investigation of the Relaxation Phenomena in Amorphous Lyophilized Solids. Pharmaceutics 2021, 13, 1735 10.3390/pharmaceutics13101735. 34684028 PMC8538343

[ref5] Cicerone M. T.; Soles C. L. Fast dynamics and stabilization of proteins: binary glasses of trehalose and glycerol. Biophys. J. 2004, 86, 3836–3845. 10.1529/biophysj.103.035519. 15189880 PMC1304285

[ref6] Cicerone M. T.; Douglas J. F. β-Relaxation governs protein stability in sugar-glass matrices. Soft Matter 2012, 8, 2983 10.1039/c2sm06979b.

[ref7] Grasmeijer N.; Stankovic M.; Waard H. de; Frijlink H. W.; Hinrichs W. L. J. Unraveling protein stabilization mechanisms: vitrification and water replacement in a glass transition temperature controlled system. Biochim. Biophys. Acta 2013, 1834, 763–769. 23360765 10.1016/j.bbapap.2013.01.020

[ref8] Xu Y.; Carpenter J. F.; Cicerone M. T.; Randolph T. W. Contributions of local mobility and degree of retention of native secondary structure to the stability of recombinant human growth hormone (rhGH) in glassy lyophilized formulations. Soft Matter 2013, 9, 7855 10.1039/c3sm51251g.

[ref9] Prestrelski S. J.; Tedeschi N.; Arakawa T.; Carpenter J. F. Dehydration-induced conformational transitions in proteins and their inhibition by stabilizers. Biophys. J. 1993, 65, 661–671. 10.1016/S0006-3495(93)81120-2. 7693001 PMC1225768

[ref10] Cleland J. L.; Lam X.; Kendrick B.; Yang J.; Yang T.; Overcashier D.; Brooks D.; Hsu C.; Carpenter J. F. A specific molar ratio of stabilizer to protein is required for storage stability of a lyophilized monoclonal antibody. J. Pharm. Sci. 2001, 90, 310–321. 10.1002/1520-6017(200103)90:3<310::aid-jps6>3.3.co;2-i. 11170024

[ref11] Andya J. D.; Maa Y. F.; Costantino H. R.; Nguyen P. A.; Dasovich N.; Sweeney T. D.; Hsu C. C.; Shire S. J. The effect of formulation excipients on protein stability and aerosol performance of spray-dried powders of a recombinant humanized anti-IgE monoclonal antibody. Pharm. Res. 1999, 16, 350–358. 10.1023/A:1018805232453. 10213364

[ref12] Wang B.; Tchessalov S.; Warne N. W.; Pikal M. J. Impact of sucrose level on storage stability of proteins in freeze-dried solids: I. Correlation of protein-sugar interaction with native structure preservation. J. Pharm. Sci. 2009, 98, 3131–3144. 10.1002/jps.21621. 19067418

[ref13] Wang B.; Tchessalov S.; Cicerone M. T.; Warne N. W.; Pikal M. J. Impact of sucrose level on storage stability of proteins in freeze-dried solids: II. Correlation of aggregation rate with protein structure and molecular mobility. J. Pharm. Sci. 2009, 98, 3145–3166. 10.1002/jps.21622. 19067392

[ref14] Chang L. L.; Shepherd D.; Sun J.; Ouellette D.; Grant K. L.; Tang X. C.; Pikal M. J. Mechanism of protein stabilization by sugars during freeze-drying and storage: native structure preservation, specific interaction, and/or immobilization in a glassy matrix?. J. Pharm. Sci. 2005, 94, 1427–1444. 10.1002/jps.20364. 15920775

[ref15] Fang R.; Obeidat W.; Pikal M. J.; Bogner R. H. Evaluation of Predictors of Protein Relative Stability Obtained by Solid-State Hydrogen/Deuterium Exchange Monitored by FTIR. Pharm. Res. 2020, 37, 168 10.1007/s11095-020-02897-7. 32794130

[ref16] Haeuser C.; Goldbach P.; Huwyler J.; Friess W.; Allmendinger A. Excipients for Room Temperature Stable Freeze-Dried Monoclonal Antibody Formulations. J. Pharm. Sci. 2020, 109, 807–817. 10.1016/j.xphs.2019.10.016. 31622600

[ref17] Svilenov H.; Winter G. The ReFOLD assay for protein formulation studies and prediction of protein aggregation during long-term storage. Eur. J. Pharm. Biopharm. 2019, 137, 131–139. 10.1016/j.ejpb.2019.02.018. 30818009

[ref18] Pikal M. J. Freeze-Drying of Proteins: Process, Formulation, and Stability; American Chemical Society, 1994; pp 120–133.

[ref19] Hancock B. C.; Shamblin S. L.; Zografi G. Molecular mobility of amorphous pharmaceutical solids below their glass transition temperatures. Pharm. Res. 1995, 12, 799–806. 10.1023/A:1016292416526. 7667182

[ref20] Tang X.; Pikal M. J. Design of freeze-drying processes for pharmaceuticals: practical advice. Pharm. Res. 2004, 21, 191–200. 10.1023/B:PHAM.0000016234.73023.75. 15032301

[ref21] Rieser R.; Menzen T.; Biel M.; Michalakis S.; Winter G. Systematic Studies on Stabilization of AAV Vector Formulations by Lyophilization. J. Pharm. Sci. 2022, 111, 2288–2298. 10.1016/j.xphs.2022.03.004. 35259349

[ref22] Muramatsu H.; Lam K.; Bajusz C.; Laczkó D.; Karikó K.; Schreiner P.; Martin A.; Lutwyche P.; Heyes J.; Pardi N. Lyophilization provides long-term stability for a lipid nanoparticle-formulated, nucleoside-modified mRNA vaccine. Mol. Ther. 2022, 30, 1941–1951. 10.1016/j.ymthe.2022.02.001. 35131437 PMC8815268

[ref23] Greiff D. Protein structure and freeze-drying: the effects of residual moisture and gases. Cryobiology 1971, 8, 145–152. 10.1016/0011-2240(71)90022-8. 5578881

[ref24] U.S. Food and Drug Administration. United States Pharmacopeia (USP) and National Formulary (NF), 2023.

[ref25] Sandulachi E. Water activity concept and its role in food preservation. Meridian Ingineresc; Universitatea Tehnica A Moldovei, 2012; pp 40–48.

[ref26] Gross J.; Sadowski G. Perturbed-Chain SAFT: An Equation of State Based on a Perturbation Theory for Chain Molecules. Ind. Eng. Chem. Res. 2001, 40, 1244–1260. 10.1021/ie0003887.

[ref27] Gross J.; Sadowski G. Application of the Perturbed-Chain SAFT Equation of State to Associating Systems. Ind. Eng. Chem. Res. 2002, 41, 5510–5515. 10.1021/ie010954d.

[ref28] Sogabe T.; Kawai K.; Kobayashi R.; Jothi J. S.; Hagura Y. Effects of porous structure and water plasticization on the mechanical glass transition temperature and textural properties of freeze-dried trehalose solid and cookie. J. Food Eng. 2018, 217, 101–107. 10.1016/j.jfoodeng.2017.08.027.

[ref29] Rahman S. Food Properties Handbook; CRC Press: Boca Raton, 1995.

[ref30] Zäh M.; Brandenbusch C.; Winter G.; Sadowski G. Predicting the amorphous-phase composition during lyophilization. Int. J. Pharm. 2023, 636, 122836 10.1016/j.ijpharm.2023.122836. 36940838

[ref31] Lorentz H. A. Ueber die Anwendung des Satzes vom Virial in der kinetischen Theorie der Gase. Ann. Phys. 1881, 248, 127–136. 10.1002/andp.18812480110.

[ref32] Wolbach J. P.; Sandler S. I. Using Molecular Orbital Calculations To Describe the Phase Behavior of Cross-associating Mixtures. Ind. Eng. Chem. Res. 1998, 37, 2917–2928. 10.1021/ie970781l.

[ref33] Held C. Measuring and Modeling Thermodynamic Properties of Biological Solutions. Dissertation, TU Dortmund University, Dortmund, 2012.

[ref34] Cameretti L. F.; Sadowski G. Modeling of aqueous amino acid and polypeptide solutions with PC-SAFT. Chem. Eng. Process. 2008, 47, 1018–1025. 10.1016/j.cep.2007.02.034.

[ref35] Krieg F. Influence of Kleptose® HP/HPB on the Molecular Interactions of a Monoclonal Antibody in Aqueous Formulations. Master Thesis, TU Dortmund University, Dortmund, 2020.

[ref36] Hübner M.; Lodziak C.; Do H. T. J.; Held C. Measuring and modeling thermodynamic properties of aqueous lysozyme and BSA solutions. Fluid Phase Equilib. 2018, 472, 62–74. 10.1016/j.fluid.2018.04.027.

[ref37] Dohrn S.; Luebbert C.; Lehmkemper K.; Kyeremateng S. O.; Degenhardt M.; Sadowski G. Phase behavior of pharmaceutically relevant polymer/solvent mixtures. Int. J. Pharm. 2020, 577, 119065 10.1016/j.ijpharm.2020.119065. 31988034

[ref38] Held C.; Sadowski G.; Carneiro A.; Rodríguez O.; Macedo E. A. Modeling thermodynamic properties of aqueous single-solute and multi-solute sugar solutions with PC-SAFT. AIChE J. 2013, 59, 4794–4805. 10.1002/aic.14212.

[ref39] Held C.; Neuhaus T.; Sadowski G. Compatible solutes: Thermodynamic properties and biological impact of ectoines and prolines. Biophys. Chem. 2010, 152, 28–39. 10.1016/j.bpc.2010.07.003. 20719425

[ref40] Fuchs D.; Fischer J.; Tumakaka F.; Sadowski G. Solubility of Amino Acids: Influence of the pH value and the Addition of Alcoholic Cosolvents on Aqueous Solubility. Ind. Eng. Chem. Res. 2006, 45, 6578–6584. 10.1021/ie0602097.

