# Supplementary material for: Water Activity as an Indicator for Antibody Storage Stability in Lyophilized Formulations
Source: Mol Pharm. 2025 Jan 14;22(2):918–26. doi: 10.1021/acs.molpharmaceut.4c01106 (PMC11795528; doi:10.1021/acs.molpharmaceut.4c01106)
Supplement: Supplementary file 1 — mp4c01106_si_001.pdf [file mp4c01106_si_001.pdf]

Supporting information

for

"*Water activity as an indicator for antibody storage stability in lyophilized formulations*"

Maximilian Zäh[a], Christoph Brandenbusch[a,*], Sebastian Groël[b], Gerhard Winter[b] and Gabriele Sadowski[a,*]

[a] *TU Dortmund University, Laboratory of Thermodynamics, Department of Biochemical and Chemical Engineering, Emil-Figge-Str. 70, 44227 Dortmund, Germany*

[b] *LMU Munich, Chair of Pharmaceutical Technology and Biopharmaceutics, Department of Pharmacy, Butenandtstr. 5, 81377 Munich, Germany*

[*]*corresponding authors:*

gabriele.sadowski@tu-dortmund.de
christoph.brandenbusch@tu-dortmund.de

Table S1: Overview on the formulation composition from literature

Formulation #	Composition / mg/ml				Residual moisture / %
	SUC	CD	rHA	PVP	
1	0	40	40	0	0.3
2	0	40	40	0	0.3
3	0	56	24	0	0.2
4	0	56	24	0	0.2
5	24	39.2	16.8	0	0.2
6	24	39.2	16.8	0	0.3
7	0	72	8	0	0.2
8	0	72	8	0	0.2
9	16	64	0	0	0.1
10	16	64	0	0	0.1
11	24	56	0	0	0.1
12	24	56	0	0	0.1
13	0	80	0	0	0.2
14	0	80	0	0	0.2
15	0	72	0	8	0.2
16	0	72	0	8	0.2
17	24	39.2	0	16.8	0.2
18	24	39.2	0	16.8	0.2

Formulation #	Composition / mg/ml				Residual moisture / %
	SUC	CD	rHA	PVP	
19	0	56	0	24	0.2
20	0	56	0	24	0.2
21	0	40	0	40	0.2
22	0	40	0	40	0.2
23	80	0	0	0	4
24	80	0	0	0	4.2

Table S2: nanoDSF data on the unfolding temperature of investigated formulations

**Formulation #**	T_{unfold} / °C	
	After drying	After 9 months
1	69.7	69.4
2	69.8	69.7
3	70.2	70.2
4	70.1	70.1
5	70.4	70.3
6	70.2	70.2
7	70.3	70.0
8	70.3	70.2

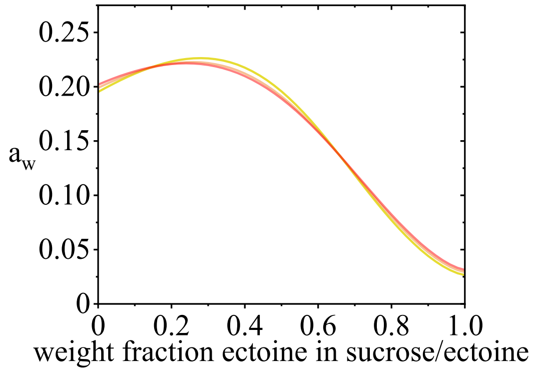

Figure S1: Water activity calculation sucrose/ectoine excipient mixture. The shown line shows the water activity with a constant glass-transition temperature of 40 °C calculated for 5 °C (yellow line), 25 °C (orange line), and 40 °C (red line).
